# Serotonergic Neurotransmission in Limbic Regions May Reflect Therapeutic Response of Depressive Patients: A PET Study With ^11^C-WAY-100635 and ^18^F-MPPF

**DOI:** 10.1093/ijnp/pyad026

**Published:** 2023-06-04

**Authors:** Soichiro Kitamura, Yasuyuki Kimura, Keisuke Takahata, Sho Moriguchi, Manabu Kubota, Hitoshi Shimada, Hironobu Endo, Yuhei Takado, Kazunori Kawamura, Ming-Rong Zhang, Tetsuya Suhara, Makoto Higuchi

**Affiliations:** Department of Psychiatry, Nara Medical University, Kashihara, Japan; Department of Functional Brain Imaging, Institute for Quantum Medical Science, National Institutes for Quantum Science and Technology, Chiba, Japan; Department of Clinical and Experimental Neuroimaging, Center for Development of Advanced Medicine for Dementia, National Center for Geriatrics and Gerontology, Obu, Japan; Department of Functional Brain Imaging, Institute for Quantum Medical Science, National Institutes for Quantum Science and Technology, Chiba, Japan; Department of Functional Brain Imaging, Institute for Quantum Medical Science, National Institutes for Quantum Science and Technology, Chiba, Japan; Department of Neuropsychiatry, Keio University School of Medicine, Tokyo, Japan; Department of Functional Brain Imaging, Institute for Quantum Medical Science, National Institutes for Quantum Science and Technology, Chiba, Japan; Department of Psychiatry, Kyoto University Graduate School of Medicine, Kyoto, Japan; Department of Functional Brain Imaging, Institute for Quantum Medical Science, National Institutes for Quantum Science and Technology, Chiba, Japan; Department of Functional Neurology & Neurosurgery, Center for Integrated Human Brain Science, Brain Research Institute, Niigata University, Niigata, Japan; Department of Functional Brain Imaging, Institute for Quantum Medical Science, National Institutes for Quantum Science and Technology, Chiba, Japan; Department of Functional Brain Imaging, Institute for Quantum Medical Science, National Institutes for Quantum Science and Technology, Chiba, Japan; Department of Radio Pharmaceutics Development, Institute for Quantum Medical Science, National Institutes for Quantum Science and Technology, Chiba, Japan; Department of Radio Pharmaceutics Development, Institute for Quantum Medical Science, National Institutes for Quantum Science and Technology, Chiba, Japan; Department of Functional Brain Imaging, Institute for Quantum Medical Science, National Institutes for Quantum Science and Technology, Chiba, Japan; Department of Functional Brain Imaging, Institute for Quantum Medical Science, National Institutes for Quantum Science and Technology, Chiba, Japan

**Keywords:** Depression, positron emission tomography, ^11^C-WAY-100635, ^18^F-MPPF, limbic lobe

## Abstract

**Background:**

Central serotonin (5-hydroxytryptamine [5-HT]) neurotransmission has been implicated in the etiology of depression. Most antidepressants ameliorate depressive symptoms by increasing 5-HT at synaptic clefts, but their effect on 5-HT receptors has yet to be clarified. ^11^C-WAY-100635 and ^18^F-MPPF are positron emission tomography (PET) radioligands for 5-HT_1A_ receptors. While binding of both ligands reflects 5-HT_1A_ receptor density, ^18^F-MPPF biding may also be affected by extracellular 5-HT concentrations. This dual-tracer PET study explored the neurochemical substrates underlying antidepressant effects in patients with depression.

**Methods:**

Eleven patients with depression, including 9 treated with antidepressants, and 16 age- and sex-matched healthy individuals underwent PET scans with ^11^C-WAY-100635 and ^18^F-MPPF. Radioligand binding was determined by calculating the nondisplaceable binding potential (*BP*_ND_).

**Results:**

Patients treated with antidepressants showed significantly lower ^18^F-MPPF *BP*_ND_ in neocortical regions and raphe nuclei, but not in limbic regions, than controls. No significant group differences in ^11^C-WAY-100635 *BP*_ND_ were found in any of the regions. Significant correlations of *BP*_ND_ between ^11^C-WAY-100635 and ^18^F-MPPF were observed in limbic regions and raphe nuclei of healthy controls, but no such associations were found in antidepressant-treated patients. Moreover, ^18^F-MPPF *BP*_ND_ in limbic regions was significantly correlated with the severity of depressive symptoms.

**Conclusions:**

These results suggest a diversity of antidepressant-induced extracellular 5-HT elevations in the limbic system among depressive patients, which is associated with the individual variability of clinical symptoms following the treatment.

**Significance Statement**

We examined central 5-HT_1A_ receptors in depressive patients and healthy controls using PET with 2 radioligands: ^11^C-WAY-100635 and ^18^F-MPPF. The binding of both ligands reflects 5-HT_1A_ receptor density, while ^18^F-MPPF binding is also susceptible to extracellular 5-HT concentrations. Unlike close correlations of the binding of these 2 tracers in control brains, anti-depressant–treated patients exhibited a lack of such correlations. This discrepancy stemmed from the low ^18^F-MPPF retentions in neocortical and raphe regions of these patients relative to controls despite no noticeable group differences in the ^11^C-WAY-100635 binding. The limbic ^18^F-MPPF binding in these antidepressant-treated patients showed diversity and was correlated with depressive symptoms, implying insufficient extracellular 5-HT elevations in this area of a subset of these cases. These findings indicate neurochemical aspects underlying the individual variability of therapeutic responses to anti-depressive agents.

## INTRODUCTION

Dysfunction of the serotonergic neurotransmission pathway in the brain has been implicated in various psychiatric disorders. Serotonin (5-hydroxytryptamine [5-HT]) neurons project from the raphe nuclei in the midbrain to broad brain areas, central to limbic regions, and modulate diverse neural activities (Courtney and Ford, 2016; Saulin et al., 2012; Smith et al., 2021). Among the 5-HT receptor subtypes, the 5-HT_1A_ receptor inhibits regulation of glutamatergic and GABAergic neurons and has been documented in psychiatric disorders (Saulin et al., 2012; Ostrowski et al., 2014; Ju et al., 2020). Functional deterioration of 5-HT_1A_ receptors has been examined in association with the pathophysiology of depression (Sargent et al., 2000; Reynolds et al., 2006; Miller et al., 2009).

^11^C-WAY-100635 (N-{2-[4-(2-methoxyphenyl)-1-piperazinyl]ethyl}-N-(2-pyridinyl)-cyclohexane carboxamide) and ^18^F-MPPF (2'-Methoxyphenyl-(N-2'-pyridinyl)-p-18F-fluoro-benzamidoethylpiperazine) are antagonistic radioligands for 5-HT_1A_ receptors and have been employed in positron emission tomography (PET) evaluation of in vivo 5-HT_1A_ availability in patients with depression (Aznavour and Zimmer, 2007; Pillai et al., 2018; Langenecker et al., 2019; Buchecker et al., 2020). Although they both selectively bind to 5-HT_1A_ receptors, ^11^C-WAY-100635 and ^18^F-MPPF are known to exhibit distinct binding properties. Because ^11^C-WAY-100635 shows a high affinity for 5-HT_1A_ receptors and displaces endogenous 5-HT bound to the target, its binding reflects 5-HT_1A_ receptor density (Hume et al., 2001; Maeda et al., 2001). Comparatively, the affinity of ^18^F-MPPF for 5-HT_1A_ receptors is lower and is close to that of endogenous 5-HT (Zhuang et al., 1994; Passchier et al., 2000). This fact raises the possibility that ^18^F-MPPF can be displaced with endogenous 5-HT, reflecting the concentrations of extracellular 5-HT (Zimmer et al., 2003; Lothe et al., 2008).

To date, there have been several reports on the in vivo evaluation of 5-HT_1A_ receptors in patients with depression. Compared with controls, some studies documented that the patients showed decreased ^11^C-WAY-100635 binding (Sargent et al., 2000; Meltzer et al., 2004; Hirvonen et al., 2008), while other studies showed increased ^11^C-WAY-100635 binding (Parsey et al., 2006; Sullivan et al., 2009; Kaufman et al., 2015). Binding of ^18^F-MPPF to 5-HT_1A_ receptors was reportedly lower in drug-naive patients with depression than in healthy controls and was elevated by administration of a selective serotonin reuptake inhibitor (SSRI) (Lothe et al., 2012). Thus, PET-assessed alterations of 5-HT_1A_ receptors in depression remain elusive. This raises the concern as to whether changes in extracellular 5-HT concentrations or 5-HT_1A_ receptor densities underlie depressive symptoms and affect PET findings.

To the best of our knowledge, no clinical examinations evaluating depression by simultaneous usage of both ^11^C-WAY-100635 and ^18^F-MPPF have been conducted. The purpose of this study was to explore alterations of the serotonergic system by PET with dual 5-HT_1A_ receptor radioligands and determine their association with clinical symptoms in the same individual patients with depression. In addition to the assessment of 5-HT_1A_ receptor density by ^11^C-WAY-100635-PET, drug-induced alterations of extracellular 5-HT levels could modify the interaction of ^18^F-MPPF with the receptors, possibly eliciting uncoupling of the 2 radioligand bindings. We hypothesized that antidepressant-induced alterations of serotonergic neurotransmission in limbic regions, which are known to be associated with mood and emotion, differentially affect binding of these 2 radioligands and correlate with the amelioration of depressive symptoms.

## MATERIALS AND METHODS

### Participants

Eleven patients with depression and 16 age- and sex-matched healthy controls were included in this study. The patients were recruited from 3 affiliated psychiatric clinics. Ten of the patients met the Diagnostic and Statistical Manual of Mental Disorders, Fourth Edition, Text Revision (DSM-IV-TR) criteria for major depressive disorder, and 1 met the criteria for dysthymic disorder. For the patients, lifetime psychiatric disorders were evaluated based on the Mini-International Neuropsychiatric Interview. Exclusion criteria included other psychiatric disorders, existence of current suicidal ideation, severe current physical illness, or administration of antidepressant drugs other than SSRIs or serotonin noradrenalin reuptake inhibitors (SNRIs). Of the 11 patients, 6 concurrently received treatment with SSRIs (20 mg/day paroxetine for 2 patients and 50–75 mg/day sertraline for 4 patients). The other 3 patients received treatment with an SNRI (30–60 mg/day duloxetine). As described elsewhere (Pae and Patkar, 2007), repeated daily dosage led to a steady state of the plasma drug concentrations long before PET scans. The remaining 2 patients were antidepressant naive. The controls were free of any somatic, neurological, or psychiatric disorders. We evaluated the severity of the depressive symptoms of all participants using the Beck Depression Inventory–II (BDI-II) (Beck et al., 1996). The patients were also evaluated by the Japanese version of the 17-item Hamilton Depression Rating Scale (HAM-D) (Hamilton, 1960). We excluded 3 healthy controls from the statistical analysis of the image data because they showed depressive tendencies with BDI-II scores exceeding 10 points (supplementary Figure 1). This study was approved by the Radiation Drug Safety Committee and the Institutional Review Board of the National Institute of Radiologic Sciences of Japan. Written informed consent was obtained from all participants. The study was registered with the University Hospital Medical Information Network Clinical Trials Registry (UMIN000012050).

### Brain PET Scans

We used ^11^C-WAY-100635 (228.3 ± 10.8 MBq, molar activity (A_m_): 147.8 ± 100 GBq/μmol) for assessing 5-HT_1A_ receptor density. We also employed ^18^F-MPPF (186.4 ± 96 MBq, A_m_: 174.8 ± 6.5 GBq/μmol) for evaluating extracellular 5-HT concentration in addition to receptor density. Out of 27 participants, 25 individuals received PET scans first with ^18^F-MPPF and then with ^11^C-WAY-100635, whereas 1 healthy control and 1 patient with depression underwent 2 PET scans in reverse order. The mean interval between the 2 PET sessions was 18 days, ranging from 7 to 68 days. After i.v. injection, 3-dimensional dynamic images were acquired using a PET scanner (Eminence SET-3000GCT/X; Shimadzu, Kyoto, Japan) for 90 and 60 minutes for ^11^C-WAY-100635 and ^18^F-MPPF, respectively. All PET images were reconstructed using the filtered back-projection method (Gaussian filter, kernel 5 mm; reconstructed in-plane resolution, 7.5 mm full width at half maximum; voxel size, 2 × 2 × 2.6 mm) with correction for attenuation, randoms, and scatter. Head motion during the scans was corrected on the emission images after attenuation correction with μ-maps realigned to each frame of the emission images (Wardak et al., 2010).

### Brain Imaging Analyses

**Figure 1.**
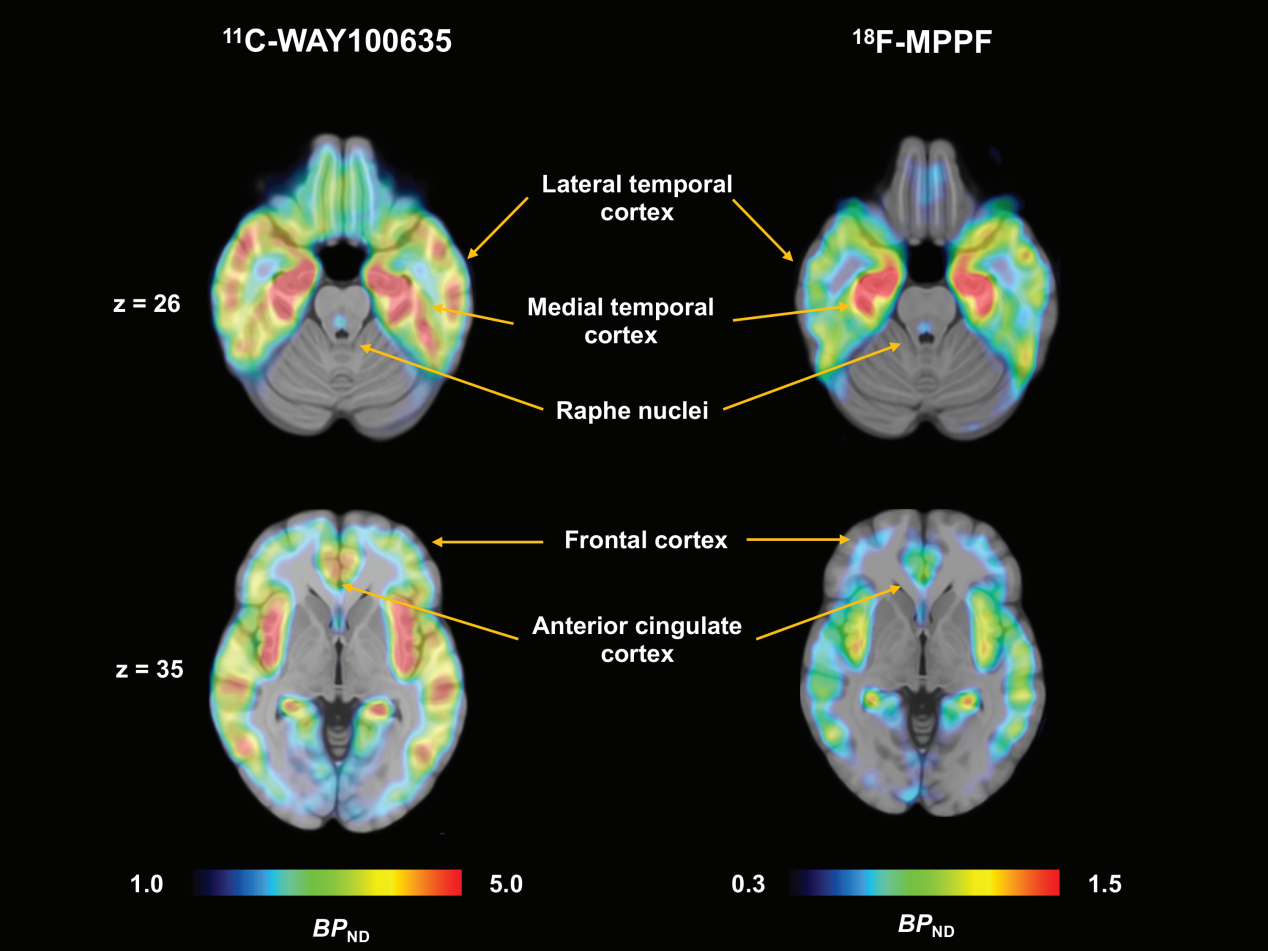
Representative parametric positron emission tomography (PET) images for nondisplaceable binding potentials (*BP*_ND_) of ^11^C-WAY-100635 and ^18^F-MPPF. The brains were axially sectioned at 2 different levels. The indicated coordinates are based on the standard MNI space.

T1-weighted magnetic resonance (MR) images were acquired using a 3-T MR imaging scanner (Vero, Siemens, Germany; TE 1.9 mms, TR 2300 mms, TI 900 mms, flip angle 9°, field of view 250 mm, acquisition matrix 256 × 256, slice thickness 1 mm). Dynamic PET data were spatially normalized based on the transformation parameters from the MR images, and time activity curves in cerebellar gray matter, receptor-poor regions, were acquired from both radioligands. The mask of cerebellar gray matter was defined with a probabilistic cerebellar atlas excluding the vermis (Diedrichsen et al., 2009). We then estimated the nondisplaceable binding potential (*BP*_ND_) using a multilinear reference tissue model (MRTM2) (Ichise et al., 2003), with the cerebellar gray matter as a reference region in the naive PET data (Hahn et al., 2010). There are also previous studies defining a reference region in cerebellar gray matter (Hirvonen et al., 2007, 2008) based on a finding of a single individual exhibiting marked radioligand retention in cerebellar gray matter. Another report demonstrated no significant difference in 5-HT_1A_ binding between the uses of cerebellar gray and white matter references (Hahn et al., 2010). We chose cerebellar gray matter as reference tissue in consideration of the substantial difference in the radioligand delivery to the brain tissue between gray and white matters. We applied a multilinear reference tissue model to the dynamic data of ^11^C-WAY-100635 because it is a suitable reference tissue approach for *BP*_ND_ estimation of ^11^C-WAY-100635 (Zanderigo et al., 2013). For this analysis, *k*_2_ʹ was calculated as the average of *k*_2_’ values determined with MRTM in the insula, hippocampus, and parahippocampal gyrus, which are enriched with the target receptors. The cerebellum was employed as a receptor-less region. Because the *BP*_ND_ value was comparably small, we applied Motmot to the *BP*_ND_ estimation of ^18^F-MPPF for good linear fitting (Ichise et al., 2003). Each of the parametric images was reregistered to the individual MR images, then spatially normalized using the Diffeomorphic Anatomical Registration Through Exponentiated Lie Algebra algorithm included in the statistical parametric mapping package (SPM12; Wellcome Trust Centre for Neuroimaging, London, UK) (Figure 1). Based on the normalized *BP*_ND_ images, we calculated the mean *BP*_ND_ values in neocortical regions, including the frontal and lateral temporal cortices, which postsynaptically express 5-HT_1A_ receptors at a relatively low level. *BP*_ND_ was also determined in limbic regions, including the medial temporal and anterior cingulate cortices, which are enriched with postsynaptic 5-HT_1A_ receptors. In addition, we calculated *BP*_ND_ in raphe nuclei, which possess presynaptic 5-HT_1A_ receptors. Four cortical regions of interest (ROIs) were defined by the automated anatomical labeling atlas (Tzourio-Mazoyer et al., 2002). Each ROI intersected with the patients’ normalized gray matter masks. Because the raphe nuclei could not be differentiated from surrounding tissue on MR images, a circular ROI 6 mm in diameter was placed on 5 contiguous axial slices to be centered at the highest radio signal in the dorsal midbrain. The ROI size was determined in light of the actual diameter of the median and dorsal raphe nuclei approximating 5 mm and the spatial resolution of the reconstructed PET images (7.5 mm full width at half maximum) in reference to previous work (Kranz et al., 2012). Analyses of all PET data were conducted using PMOD 3.7 (PMOD Technologies Ltd.).

### Statistical Analyses

For the statistical analyses, we excluded 2 depressive patients without antidepressant treatment to focus on the treatment-related neuroimaging changes (supplementary Figure 1) and displayed data from these individuals in the graph plots for preliminary comparisons. Unpaired *t* tests were used to evaluate group comparisons with demographic parameters of age, BDI-II scores, and Fisher’s exact test for sex (statistical significance: *P* < .05). We performed unpaired *t* tests to examine the group differences in the estimated *BP*_ND_ values of either ^11^C-WAY-100635 or ^18^F-MPPF between the patients and healthy controls. To evaluate the mutual relationship between the estimated *BP*_ND_ values of ^11^C-WAY-100635 and ^18^F-MPPF, correlation analyses were performed. Among the patients with depression, we evaluated the relationships between severity of depression by HAM-D scores and the estimated *BP*_ND_ values of both radioligands, respectively. Because estimated *BP*_ND_ values of ^11^C-WAY-100635 and ^18^F-MPPF, and HAM-D scores showed normal distribution as examined by the Shapiro-Wilk test, correlations between these parameters were examined by *t* test of Pearson correlation coefficient (statistical significance: *P* < .05). Statistical analyses were conducted using the Statistical Package for the Social Sciences (SPSS) version 22 (SPSS Inc., Chicago, IL, USA).

## RESULTS

### Demographic Characteristics

The demographic and psychological profiles of the participants in this study are shown in Table 1. There were no significant differences in age or gender between the patients and healthy controls. The patients showed significantly higher BDI-II scores than those of the healthy controls (*P* < .05).

**Table 1.** Demographic Characteristics of the Current Participants

	Healthy Controls (n = 16)	Depressive Patients (n = 11)	*t* or χ^2^	*P*
Age, mean (SD), y	34.6 (9.7)	35.3 (5.4)	-0.22	.83
Sex, male (%)	8 (50)	4 (36)	0.49	.70
Duration of illness, mean (SD)	N/A	19.9 (16.9)	N/A	N/A
HAM-D, mean (SD)	N/A	12.5 (6.09)	N/A	N/A
BDI-II, mean (SD)	6.31 (5.2)	25.4 (8.5)	−7.1	<.001^*^

BDI, Beck Depression inventory-II; HAM-D, Hamilton depression rating scale.

^*^ *P* < .05.

### Group Comparisons of ^*11*^C-WAY-100635 and ^*18*^F-MPPF

**Figure 2.**
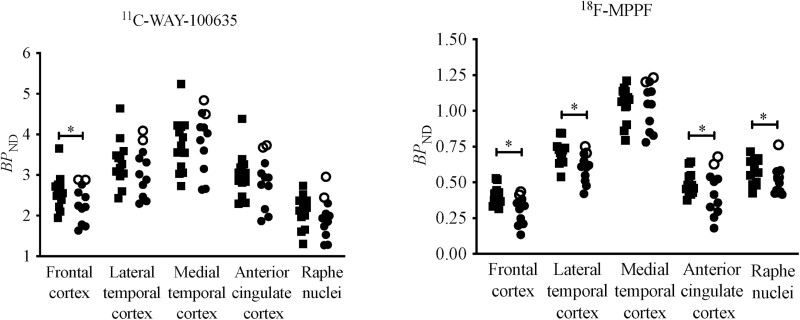
Group comparisons of the estimated nondisplaceable binding potential (*BP*_ND_) values of both ^11^C-WAY-100635 and ^18^F-MPPF between patients with depression and healthy controls. Filled squares indicate the healthy controls, filled circles indicate the patients treated with antidepressants, and open circles indicate depressive patients without such medication. There was significantly lower binding of ^11^C-WAY-100635 in the frontal cortex of the antidepressant-treated patients compared with controls (left; *P* < .05). On the other hand, the patients showed significantly lower estimated *BP*_ND_ values of ^18^F-MPPF in the frontal, lateral temporal, and anterior cingulate cortices and raphe nuclei than the healthy controls (right; *P* < .05).

*BP*_ND_ values of ^11^C-WAY-100635 and ^18^F-MPPF in the patients versus healthy controls are shown in Figure 2 and supplementary Table 1. Significantly lower ^11^C-WAY-100635 *BP*_ND_ values were seen in the frontal cortex of the antidepressant-treated patients than those of the healthy controls (*P* < .05). By contrast, the antidepressant-treated patients exhibited lower ^18^F-MPPF *BP*_ND_ values in the frontal, lateral temporal, and anterior cingulate cortices and raphe nuclei than the healthy controls (*P* < .05). These group differences in the ^18^F-MPPF binding remained statistically significant in the frontal and lateral temporal cortices and raphe nuclei after applying Bonferroni’s correction for multiple comparisons (*P* < .01).

### Mutual Relationships Between ^*11*^C-WAY-100635 and ^*18*^F-MPPF Binding

**Figure 3.**
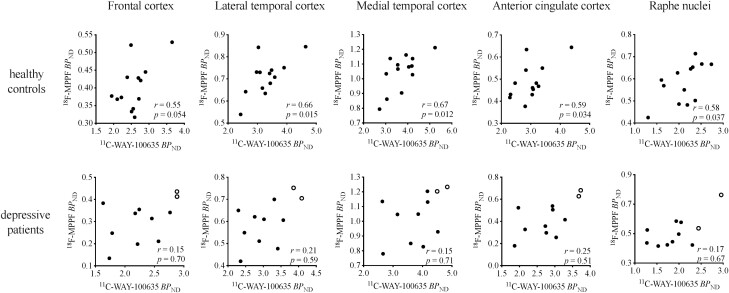
The relationship between the estimated nondisplaceable binding potential (*BP*_ND_) values of ^11^C-WAY-100635 and ^18^F-MPPF among patients with depression and healthy controls. Among the patients, filled circles indicate those treated with antidepressants and open circles those untreated. There were significant positive correlations between the 2 radioligand bindings in the lateral and medial temporal and anterior cingulate cortices and raphe nuclei of the healthy controls (*P* < .05). By contrast, no such correlations were observed in any of the brain regions of the antidepressant-treated patients (*P* > .05).

Figure 3 demonstrates the mutual relationships between the estimated *BP*_ND_ values of ^11^C-WAY-100635 and ^18^F-MPPF in each of the 2 groups. Significant positive correlations between the 2 radioligand bindings were observed in all brain regions (lateral temporal cortex, *r* = 0.66, *P* = .015; medial temporal cortex, *r* = 0.67, *P* = .012; anterior cingulate cortex, *r* = 0.59, *P* = .034; raphe nuclei, *r* = 0.58, *P* = .037) of the healthy controls except the frontal cortex (*r* = 0.55, *P* = .054). However, no brain regions in the antidepressant-treated patients showed such significant correlations (frontal cortex, *r* = 0.15, *P* = .70; lateral temporal cortex, *r* = 0.21, *P* = .59; medial temporal cortex, *r* = 0.15, *P* = .71; anterior cingulate cortex, *r* = 0.25, *P* = .51; raphe nuclei, *r* = 0.17, *P* = .67).

### Associations Between Severity of Depressive Symptoms and Radioligand Binding

**Figure 4.**
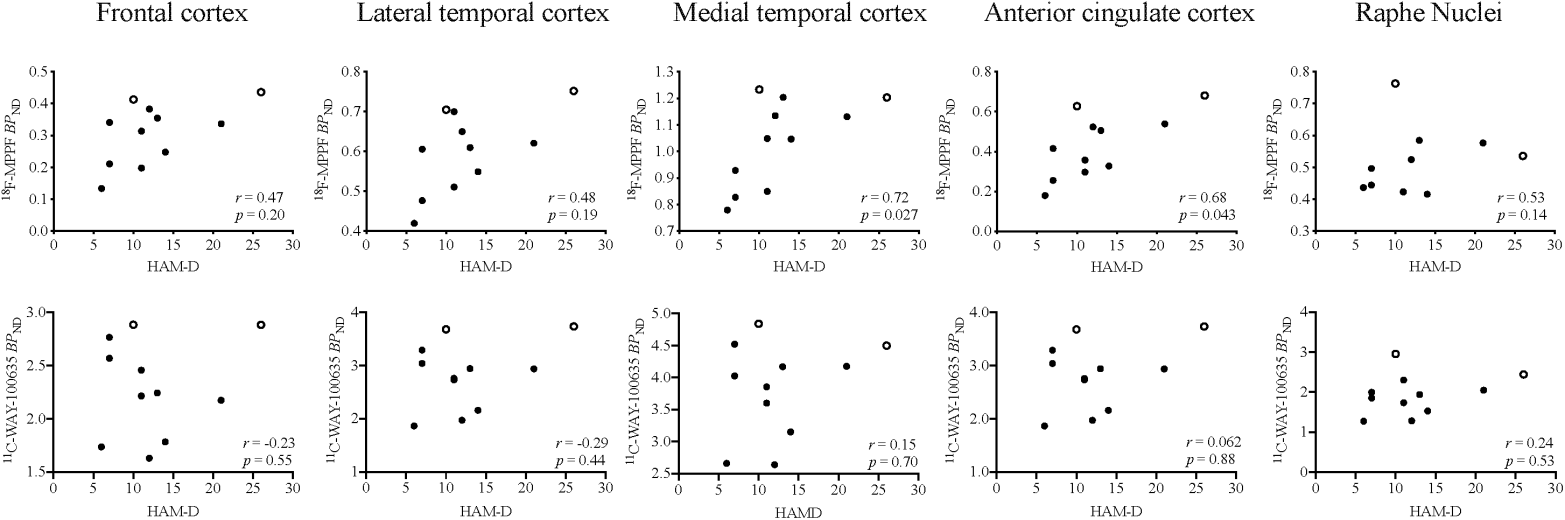
The relationship between the estimated ^18^F-MPPF nondisplaceable binding potential (*BP*_ND_) values and the severity of depression according to Hamilton depression rating scale (HAM-D) scores among patients with depression. Filled and open circles indicate treated and untreated patients, respectively. There were significant positive correlations between HAM-D scores and estimated ^18^F-MPPF *BP*_ND_ values in the medial temporal and anterior cingulate cortices of the antidepressant-treated cases (*P* < .05). No significant correlations were observed in other regions (*P* > .05). The estimated ^11^C-WAY-100635 *BP*_ND_ values had no association with disease severity in any of the brain regions (*P* > .05).

Significant positive correlations were also noted between HAM-D scores and the estimated ^18^F-MPPF *BP*_ND_ values in limbic regions, such as the medial temporal and anterior cingulate cortices, of the antidepressant-treated patients (medial temporal cortex, *r* = 0.72, *P* = .027; anterior cingulate cortex, *r* = 0.68, *P* = .043). No significant correlations were noted in neocortical and brainstem regions (frontal cortex, *r* = 0.47, *P* = .20; lateral temporal cortex, *r* = 0.48, *P* = .19; raphe nuclei, *r* = 0.53, *P* = .14) (Figure 4). Unlike ^18^F-MPPF, no significant correlations between the estimated ^11^C-WAY-100635 *BP*_ND_ values and HAM-D scores in any of the regions examined were found (frontal cortex, *r* = -0.23, *P* = .55; lateral temporal cortex, *r* = -0.29, *P* = .44; medial temporal cortex, *r* = 0.15, *P* = .70; anterior cingulate cortex, *r* = 0.062, *P* = .88; raphe nuclei, *r* = 0.24, *P* = .53).

## DISCUSSION

To the best of our knowledge, this is the first study to compare the binding of the 2 PET radioligands for 5HT_1A_ receptors in patients with depression. There were no differences in ^11^C-WAY-100635 *BP*_ND_ values between patients with depression and healthy controls in most of the brain regions, implying that there were no marked changes in the density of 5-HT_1A_ receptors. However, ^18^F-MPPF *BP*_ND_ values in the neocortex and raphe nuclei of antidepressant-treated patients were significantly lower than in controls (Figure 2). ^11^C-WAY-100635 and ^18^F-MPPF exert different properties when binding to 5-HT_1A_ receptors. The binding of both ligands is considered to reflect 5-HT_1A_ receptor density, while ^18^F-MPPF binding could also be influenced by extracellular 5-HT molecules. Accordingly, the positive correlations between the *BP*_ND_ values of ^11^C-WAY-100635 and ^18^F-MPPF in most brain areas of healthy controls (Figure 3) suggest that PET data with both radioligands provide indicators for the density of 5-HT_1A_ receptors under a normal physiological condition. Meanwhile, the correlation between the 2 radioligand bindings appeared to be weakened in the antidepressant-treated patients. To ensure this finding, we statistically examined the difference in the coefficients of these correlations across brain regions between the patients and controls by unpaired *t* test and found that the coefficient values were significantly smaller in the patients than in the controls (*P* = .05; supplementary Figure 2), conceivably resulting from drug-induced increases in extracellular 5-HT levels and consequent decreases in ^18^F-MPPF *BP*_ND_.

It is noteworthy that the difference in ^18^F-MPPF binding between the treated depressive cases and controls was less remarkable in the medial temporal and anterior cingulate cortices, indicating that limbic extracellular 5-HT levels might not be markedly elevated in response to the treatment in a significant subset of patients. This was previously documented in the literature (Van Dyke et al., 2019; Pu et al., 2021). This finding could be associated with the efficacy of antidepressants, because patients with higher limbic ^18^F-MPPF binding, which might stem from less profound drug-induced increases in 5-HT levels, exhibited higher scores on the depressive scale. Hence, the serotonergic system in the limbic region of patients with depression differentially responded to anti-depressive agents. This appears to be critically linked to the severity of depression during treatment.

Notably, the present study demonstrated that *BP*_ND_ of ^18^F-MPPF in limbic regions, especially the medial temporal cortex, exhibited significant correlations with the severity of depression. Limbic regions, including the medial temporal and anterior cingulate cortices, have been known to contain abundant 5-HT_1A_ receptors and diminished serotonergic neurotransmissions in these regions have been implicated in depressive symptoms (Saulin et al., 2012;Wang et al., 2016). Our findings suggest that a marked response to antidepressant medication may be elicited by a noticeable increase in extracellular 5-HT, which is represented by relatively low ^18^F-MPPF binding to 5-HT_1A_ receptors. The Sequenced Treatment Alternatives to Relieve Depression trial indicated that a proportion of patients were resistant to sequential antidepressant treatments, highlighting the need for an objective indicator of drug efficacies at an initial stage of therapy (Rush et al., 2006). The current findings also imply that evaluation of 5-HT status in limbic regions using ^18^F-MPPF early during therapeutic intervention in serotonergic transmission serves as a predictor of subsequent effects of individual symptoms. Accordingly, those who are potentially resistant to 5-HT-enhancing agents may be engaged by other approaches exemplified by augmentation therapies and non-drug treatments.

The reduction of 5-HT neurotransmission is critically involved in the molecular and neurochemical etiology of depression, which causes upregulation of postsynaptic 5-HT_1A_ receptors (Albert, 2012; Underwood et al., 2012). Previous studies have reported that untreated depressive patients with depression without antidepressant medication showed elevated 5-HT_1A_ receptor binding of ^11^C-WAY-100635 relative to healthy controls (Parsey et al., 2006; Metts et al., 2019). Accordingly, the 2 depressive patients without antidepressant medication examined in this study showed a tendency of high ^11^C-WAY-100635 *BP*_ND_ compared with healthy controls in each of the brain regions. Antidepressant treatment appears to enhance synaptic 5-HT neurotransmission, resulting in reversal of the synaptic 5-HT_1A_ receptor density as assessed by ^11^C-WAY-100635. Parsey and colleagues also reported no significant differences in ^11^C-WAY-100635 binding between combined antidepressant-treated and untreated patients with depression and healthy controls (Parsey et al., 2006). Despite the putative antidepressant-induced normalization of 5-HT_1A_ receptor density, there were no significant correlations between the estimated ^11^C-WAY-100635 *BP*_ND_ values and the severity of the depressive symptoms in treated patients. This indicates that the density of 5-HT_1A_ receptors assessed by ^11^C-WAY-100635-PET may not offer a neurochemical index of therapeutic efficacy.

In raphe nuclei, ^18^F-MPPF binding in patients with depression treated with antidepressants was significantly lower than that of healthy controls. 5-HT_1A_ autoreceptors mainly distribute to the raphe nuclei and inhibit synaptic 5-HT release (Zimmer et al., 2004; Courtney and Ford, 2016). Antidepressant medication induces 5-HT_1A_ autoreceptor internalization, presumably reducing ^18^F-MPPF-accessible receptors. Previous studies have reported that antidepressant administration can be associated with 5-HT_1A_ autoreceptor internalization in raphe nuclei, which was consistent with the current findings (Riad et al., 2004; Sibon et al., 2008). Depressive severity was not associated with the estimated *BP*_ND_ values of the 2 radioligands in this area. As autoreceptor internalization in raphe nuclei may not directly reflect synaptic 5-HT concentrations in the projection areas, the relationship between depressive symptoms and 5-HT_1A_ receptor alterations may be elusive.

Several limitations need to be considered in this study. First, the statistical significance of the correlations between the 2 radioligand bindings and between the limbic ^18^F-MPPF binding and HAM-D scores did not survive Bonferroni correction for multiple comparisons, primarily due to the relatively small sample size. However, such corrections are usually of importance for examining a single positive finding among many negative results. By contrast, a large portion of the *t* tests and correlation analyses performed here showed uncorrected significance. Nonetheless, the positive findings in the correlational analysis might be considered trends. Second, the number of antidepressant-naive and remitted patients was insufficient. Third, because the patients showed mainly mild or moderate disease severity, 5-HT_1A_ receptor density and extracellular 5-HT concentration in severely depressive and antidepressant-resistant patients are yet to be investigated. Because the severity of depressive symptoms was assessed at the time of the first PET scan, there was a possibility of minor mood fluctuations towards the second PET scan, notwithstanding the continuous treatment during this period and the relatively short interval (<2 weeks) between the 2 scans. The current data could also be affected by several factors other than antidepressant treatments, including nonpharmacological therapy given to the treated patients for a certain period as the participants of this study were not evaluated by PET with ^11^C-WAY-100635 and ^18^F-MPPF before antidepressant administration.

Additionally, most patients with depression in the current study received antidepressant medication at the time of the PET scans, impeding the separation between the disease- and treatment-associated alterations of the 5-HT_1A_ radioligand binging. The present assay was conducted under the assumption that the disease-associated changes in the receptor density could be evaluated by PET with ^11^C-WAY-100635 because the binding of this radioligand is not susceptible to drug-induced elevations of extracellular 5-HT concentrations. However, this issue would be preferably addressed by PET assays of drug-naïve patients. Despite no direct interaction between antidepressants used in this study (SSRI or SNRI) and 5-HT_1A_ receptors, indirect influences of these drugs on the receptors via enhanced stimulation by 5-HT would be more appropriately identified by analyzing ^18^F-MPPF binding before and after the initiation of the antidepressant therapy, although the performance of PET in untreated cases with depression might cause a delay in the commencement of the therapy. The antidepressant-provoked change in ^18^F-MPPF binding and its association with clinical outcome should also be assessed, preferably in a longitudinal study consisting of examinations at baseline, as well as during treatment. Such analysis would be conducted with a larger sample size to assess the influences of the antidepressant types (e.g., SSRI vs SNRI) on PET findings. Moreover, we did not obtain detailed information on the histories of smoking, drinking, and intake of drugs other than antidepressants, although we briefly confirmed that each healthy control was neither a heavy smoker nor a heavy alcohol consumer upon the visit to the PET facility. Accordingly, we did not consider these factors as determinants of the exclusion criteria or confounding parameters in the statistical analysis.

This dual radioligand study was conducted based on the viewpoint that ^18^F-MPPF binding reflected extracellular 5-HT concentrations as well as 5-HT_1A_ receptor density, in contrast to ^11^C-WAY100635 binding solely reflecting receptor density. Because the inhibition constant (K_i_) of ^18^F-MPPF for 5-HT_1A_ receptors is 3.3 nM, much higher than that of ^11^C-WAY100635 (0.8 nM) (Zhuang et al., 1994), ^18^F-MPPF binding is more susceptible to extracellular 5-HT levels than ^11^C-WAY100635 (Zimmer et al., 2002). We could not entirely rule out the possibility that the difference in binding potentials between the 2 ligands may arise from the reactivity of these compounds to off-target molecules, although WAY100635 and MPPF are known to display >100-fold and 50-fold selectivity, respectively, for 5-HT_1A_ receptors relative to other diverse CNS receptors, including non-1A 5-HT receptor subtypes (Gozlan et al., 1995; Kung et al., 1996). It is also likely that antidepressant treatment alters the nondisplaceable retention of ^18^F-MPPF. Furthermore, 5-HT_1A_ receptors may be present in both high-affinity and low-affinity states (Watson et al., 2000), with the ratio between these states presumably being susceptible to changes in extracellular 5-HT levels caused by an antidepressant. ^18^F-MPPF could also have higher selectivity for the receptors in the high-affinity state than ^11^C-WAY100635. Besides these possibilities, it should also be considered that the drug-induced increase in extracellular 5-HT could promote internalization of the receptor, and ^18^F-MPPF might not readily access the internalized receptors (Udo de Haes et al., 2005), unlike ^11^C-WAY100635 (Gozlan et al., 1995). If ^18^F-MPPF binding is affected by the affinity state and internalization of 5-HT_1A_ receptors elicited by 5-HT reuptake inhibition, the binding may still provide an indirect index for the extracellular 5-HT levels.

In conclusion, the combined use of ^11^C-WAY-100635 and ^18^F-MPPF can facilitate detailed in vivo assessments of the serotonergic system in depression. Serotonergic alterations in neocortical regions and raphe nuclei had less association with the severity of depressive symptoms. Serotonergic dysfunction in limbic regions is suggested to be critically significant in the pathophysiology of depression and the therapeutic effects of antidepressant treatment. Because adequate 5-HT release, induced by antidepressant medication in limbic regions, is clinically important for the improvement of depression, ^18^F-MPPF binding in limbic regions may enable evaluation of the neurochemical responses to psychotropic treatment.

## Supplementary Material

pyad026_suppl_Supplementary_Figure_S1

pyad026_suppl_Supplementary_Figure_S2

pyad026_suppl_Supplementary_Table_S1

## Data Availability

Data available on request.
