# Supplementary material for: Serotonergic Neurotransmission in Limbic Regions May Reflect Therapeutic Response of Depressive Patients: A PET Study With 11C-WAY-100635 and 18F-MPPF
Source: Int J Neuropsychopharmacol. 2023 Jun 4;26(7):474–82. doi: 10.1093/ijnp/pyad026 (PMC10388381; doi:10.1093/ijnp/pyad026)
Supplement: pyad026_suppl_Supplementary_Figure_S1 [file pyad026_suppl_supplementary_figure_s1.docx]

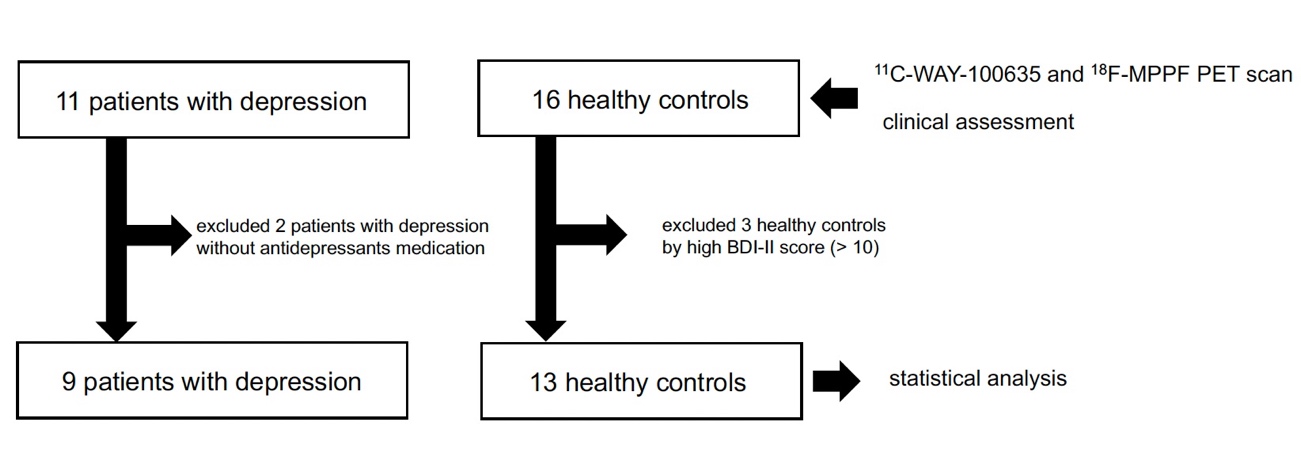


Supplementary Figure S1. Flow chart of inclusion for statistical analysis in patients with depression and healthy controls.

BDI-II, Beck Depression Inventory–II
