# Supplementary material for: Serotonergic Neurotransmission in Limbic Regions May Reflect Therapeutic Response of Depressive Patients: A PET Study With 11C-WAY-100635 and 18F-MPPF
Source: Int J Neuropsychopharmacol. 2023 Jun 4;26(7):474–82. doi: 10.1093/ijnp/pyad026 (PMC10388381; doi:10.1093/ijnp/pyad026)
Supplement: pyad026_suppl_Supplementary_Figure_S2 [file pyad026_suppl_supplementary_figure_s2.doc]

Supplementary Figure S2.　Group comparison of the regional correlation coefficients of estimated *BP*_ND_ between ^11^C-WAY-100635 and ^18^F-MPPF. Calculation of the *t*-test confirmed that healthy controls showed significantly higher regional correlation coefficients than patients with depression (healthy controls, 0.61 ± 0.05; patients with depression, 0.19 ± 0.05; T = 13.6; *p* < 0.001).

**p* < 0.05

*BP*_ND_, non-displaceable binding potential
