# Supplementary material for: Serotonergic Neurotransmission in Limbic Regions May Reflect Therapeutic Response of Depressive Patients: A PET Study With 11C-WAY-100635 and 18F-MPPF
Source: Int J Neuropsychopharmacol. 2023 Jun 4;26(7):474–82. doi: 10.1093/ijnp/pyad026 (PMC10388381; doi:10.1093/ijnp/pyad026)
Supplement: pyad026_suppl_Supplementary_Table_S1 [file pyad026_suppl_supplementary_table_s1.docx]

| Supplementary Table S1. Group comparisons of the estimated *BP*_ND_ values of ^11^C-WAY-100635 and ^18^F-MPPF. | | | | |
| --- | --- | --- | --- | --- |
|  | healthy subjects | depressive patients | *t* | *p* |
| ^11^C-WAY-100635 |  |  |  |  |
| Frontal cortex | 2.58 (0.42) | 2.17 (0.39) | 2.26 | 0.035^†^ |
| Lateral temporal cortex | 3.31 (0.56) | 2.90 (0.47) | 1.80 | 0.087 |
| Medial temporal cortex | 3.75 (0.67) | 3.65 (0.68) | 0.34 | 0.737 |
| Anterior cingulate cortex | 3.00 (0.54) | 2.63 (0.63) | 1.60 | 0.125 |
| Raphe nuclei | 2.11 (0.40) | 1.77 (0.35) | 2.04 | 0.370 |
| ^18^F-MPPF |  |  |  |  |
| Frontal cortex | 0.40 (0.07) | 0.28 (0.08) | 3.88 | 0.001^†, ††^ |
| Lateral temporal cortex | 0.71 (0.08) | 0.57 (0.09) | 3.70 | 0.001^†, ††^ |
| Medial temporal cortex | 1.05 (0.12) | 1.00 (0.15) | 0.86 | 0.402 |
| Anterior cingulate cortex | 0.49 (0.08) | 0.38 (0.13) | 2.50 | 0.021^†^ |
| Raphe nuclei | 0.58 (0.09) | 0.48 (0.07) | 2.89 | 0.009^†, ††^ |

Values are listed as mean (SD).

†; *p* < 0.05.

††; *p* < 0.01.
